# The multi-objective optimization of residential building glass in summer-hot and winter-cold regions using genetic algorithms: energy consumption, carbon emissions, and health performance analysis

**DOI:** 10.3389/fpubh.2025.1606590

**Published:** 2026-01-13

**Authors:** Yuanyuan He, Xin Fu, Shuo Li, Jie Guo

**Affiliations:** 1 College of Art and Design, Wuhan Textile University, Wuhan, China; 2 College of Chemistry and Chemical Engineering, Wuhan Textile University, Wuhan, China

**Keywords:** genetic algorithm, high-performance glass, multi-objective optimization, energy consumption, carbon emissions, health performance

## Abstract

**Introduction:**

With the growing demand for environmental sustainability and residential comfort, low-carbon buildings and healthy urban planning have become key research priorities. In summer-hot and winter-cold regions, the performance of residential building glazing plays a critical role in balancing energy efficiency, carbon emissions, and indoor health.

**Methods:**

This study investigates the multi-objective optimization of residential building glass using genetic algorithms. Building energy consumption, carbon emissions, and indoor health performance are set as optimization objectives. Key glass parameters—including the window heat transfer coefficient, solar heat gain coefficient, and visible light transmittance—are optimized through a multi-objective genetic algorithm framework. Simulations are conducted using the Rhino and Grasshopper platforms, with Hangzhou, China, selected as the case study area.

**Results:**

The optimization results indicate that annual building energy consumption decreases from 50.95 to 40.26 kWh/(m^2^·a), representing a reduction of 20.98%. Carbon emissions are reduced from 2622.93 to 2083 kgCO_2_e/m^2^, a decrease of 20.57%. In addition, the proportion of indoor healthy time increases from 34.46% to 40.9%, corresponding to an improvement of 18.69%.

**Discussion:**

By comprehensively considering energy efficiency, carbon emissions, and indoor health performance, this study proposes an optimized glazing configuration for residential buildings in summer-hot and winter-cold regions. The results suggest prioritizing south-facing windows in building design, while adjusting glass parameters for other orientations according to specific conditions. This work provides practical technical support and optimization strategies for the development of low-carbon buildings and healthy cities.

## 1 Introduction

As the issue of global climate change becomes increasingly severe, the building industry, as a major sector of energy consumption and carbon emissions, faces enormous challenges. The international community has reached a broad consensus. For example, the United Nations Sustainable Development Goals clearly require ensuring universal access to affordable and sustainable energy, and building inclusive, safe, resilient, and sustainable cities and communities. International policies such as the European Union’s *Energy Performance of Buildings Directive* also strongly promote the development of the construction sector toward near-zero energy consumption and whole-life-cycle carbon neutrality. Against this background, improving building energy efficiency and addressing climate change have become global priorities. According to statistics from the International Energy Agency, building energy consumption accounts for approximately 40% of global total energy use, with residential buildings showing significant variations in energy consumption across different seasons and climate conditions. Particularly in summer-hot and winter-cold regions, the energy consumption problem is especially prominent, as high temperatures in summer and severe cold in winter require buildings to consume substantial energy for air conditioning and heating. Traditional building designs and materials often fail to meet the requirements for low energy consumption and environmental protection (1, 2). Therefore, finding ways to reduce energy consumption while enhancing building comfort has become a critical issue in current architectural design. The core goal of low-carbon building design is to reduce energy consumption and carbon emissions during the operation of buildings through rational use of building materials and optimization of building facades and window structures, thereby achieving environmental sustainability (3). In low-carbon building design, high-performance glass is an important building material. With its excellent thermal insulation, soundproofing, and light transmission properties, it has gradually become one of the key materials for improving building energy efficiency and comfort. High-performance glass can effectively reduce heat conduction and radiation, and lower building energy consumption, particularly under extreme climate conditions where its role is particularly significant. Compared to traditional glass, low-emissivity (Low-E) glass, vacuum glass, and other high-performance glass options can significantly enhance the thermal performance of building envelopes through optimized structures and materials, and effectively improve indoor environmental quality (4, 5). At the same time, the building environment is closely related to residents’ health and public well-being. The World Health Organization points out that people spend more than 80% of their time indoors. Indoor environmental quality directly affects the occupants’ physical health, mental state, and work efficiency. A poor thermal comfort environment increases the risk of cardiovascular and respiratory diseases; insufficient or uneven lighting easily leads to visual fatigue, circadian rhythm disorders, and seasonal affective disorder; glare through windows causes problems such as headaches and decreased attention (6, 7). Therefore, optimizing the performance of building envelopes—especially window glass, which serves as the interactive interface between indoor and outdoor environments is related to energy efficiency; it is also an important issue in the fields of built environment health and public health. However, as the application of high-performance glass in building design increases, how to effectively balance and optimize various performance parameters remains a pressing challenge.

Currently, there are numerous studies on the application of high-performance glass in low-carbon building design. Mocerino (8) improved energy efficiency, reliability, and comfort through innovative glass technology using lean manufacturing and robotic technology while also reducing costs and increasing productivity. The focus included smart glass facades, hybrid hydrogen systems, and the integrated design and application of renewable energy. Khaled and Berardi (9) reviewed current glass coating technologies, and compared static and dynamic coatings, including Low-E, electrothermal, photothermal, electrochromic, gasochromic, photochromic, and thermochromic coatings. Low-E coatings have nearly reached their energy-saving limits, while photothermal coatings improve thermal performance by absorbing ultraviolet (UV) and near-infrared radiation. Dynamic coatings can adjust solar gain in response to external stimuli, though electrochromic and gasochromic coatings are relatively expensive. Taser et al. (10) explored the potential of integrating photovoltaic technology into windows to enhance the energy generation capability, thermal performance, and lighting effects of building facades. Using genetic evolutionary optimization algorithms, they optimized the design of semi-transparent amorphous silicon photovoltaic glass. The results showed that the optimized glass increased the spatial daylight autonomy to 82%, reduced the risk of glare, significantly lowered the lighting load, and simultaneously increased the annual electricity generation. However, most current studies mainly focus on the passive energy-saving effects of glass materials. There is no systematic research on their comprehensive optimization, especially in seeking the optimal balance among energy consumption, carbon emissions, and health performance.

To address this research gap, this work proposes a multi-objective optimization method for residential building glass based on the Genetic Algorithm (GA). Taking residential window glass as the research subject, a multi-objective optimization model is constructed with the goals of reducing building operational energy consumption, decreasing lifecycle carbon emissions, and enhancing indoor health performance. A typical residential building in a summer-hot and winter-cold region (Hangzhou, China) is used as a case study for glass parameter optimization, and an optimal solution is proposed. The actual effects of glass optimization are quantified through comparisons of energy consumption, carbon emission assessments, and indoor health performance analysis before and after optimization. The innovation of this work lies in using GA to realize the multi-objective optimization of residential building glass performance. It significantly improves optimization efficiency while integrating energy consumption, carbon emissions, and health performance to form a comprehensive optimization solution. Therefore, this work hypothesizes that a GA-based multi-objective optimization approach can effectively reduce both operational energy consumption and full-life-cycle carbon emissions of residential buildings in hot-summer-cold-winter regions. These improvements are achievable while maintaining satisfactory indoor health performance standards. The main objectives include establishing a multi-objective model suitable for the performance optimization of residential building glass in hot-summer and cold-winter regions. GA is used to optimize the key performance parameters of window glass. In addition, it quantitatively analyzes the effects of the optimization solution in terms of energy consumption, carbon emissions, and indoor health performance. Meanwhile, a promotable optimization method and technical path are provided for low-carbon and healthy building design. This work provides a reference for energy-efficient design in residential buildings in summer-hot and winter-cold regions and promotes the wider application of high-performance glass in the building industry, thereby contributing to the development of low-carbon, healthy buildings.

## 2 Methods

### 2.1 Analysis of factors affecting energy consumption and health performance in residential buildings in summer-hot and winter-cold regions

The climatic characteristics of summer-hot and winter-cold regions determine the unique needs of residential buildings in terms of energy consumption and health performance. In these regions, summers are hot and humid, while winters are cold and damp, with drastic temperature fluctuations throughout the year. This results in significant heating and cooling demands for buildings. Additionally, the high humidity in summer often leads to stuffy conditions, while the cold temperatures in winter cause discomfort. Therefore, the performance of the building envelope, heating and cooling systems, window characteristics, and indoor environmental control all have a significant impact on both building energy consumption and occupant health.

The thermal performance of the building envelope directly affects the heating and cooling load of the building. The insulation performance of exterior walls, roofs, and floors determines the stability of indoor temperatures. Envelope structures with low thermal conductivity can reduce heat loss in winter and cooling loss in summer, thereby lowering the energy consumption of the air conditioning system (11, 12). Moreover, the thermal mass of the exterior walls also influences the energy consumption of the building. For instance, high thermal mass walls can delay the impact of outdoor temperature fluctuations on the indoor environment, resulting in a more stable indoor temperature.

**Figure 1:**
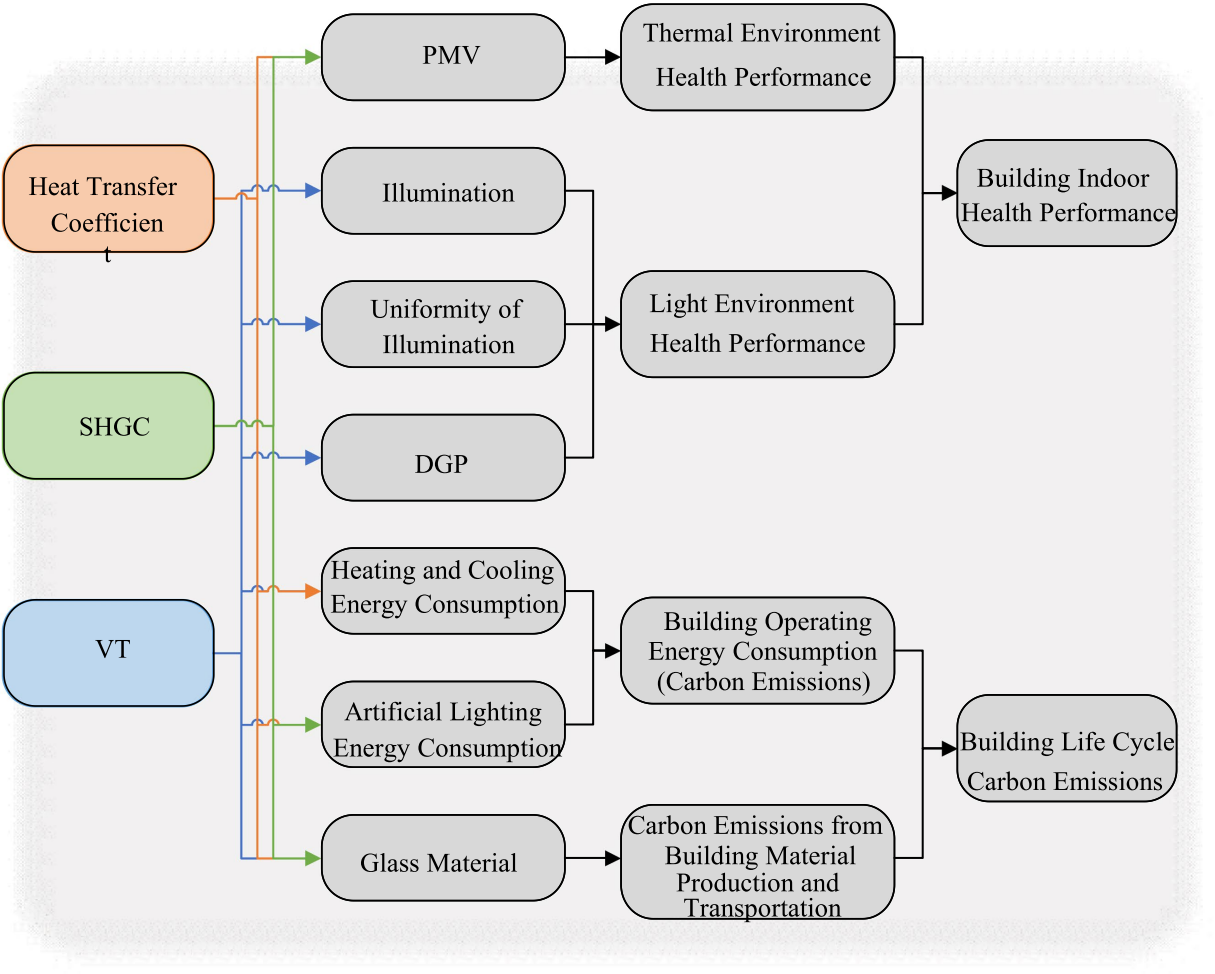
Impact pathways of glass performance parameters on residential building energy consumption and health performance.

Key indicators for assessing window energy efficiency and health performance include the main window glass’ thermal transmittance coefficient (K-value), solar heat gain coefficient (SHGC), and visible transmittance (VT) (13, 14). A lower thermal transmittance coefficient reduces heat loss in winter and heat gain in summer, improving the building’s energy efficiency. The SHGC influences the risk of overheating in summer and the use of solar energy in winter. Proper window parameters can improve indoor thermal comfort while reducing energy consumption. VT affects the quality of natural lighting indoors. If the VT is too low, it increases the need for artificial lighting during the day, raising energy consumption. If the VT is too high, it can cause glare issues, compromising visual comfort. Figure 1 illustrates the specific impact pathways.

The proper control of indoor temperature and humidity is crucial for reducing energy consumption and enhancing occupant comfort and health (15). In summer-hot and winter-cold regions, due to low winter temperatures, there is a high demand for heating, while the hot and humid environment in summer increases the burden on air conditioning, cooling, and dehumidification. Proper window performance, shading design, and natural ventilation strategies can effectively regulate the indoor thermal and humidity environment, reduce the load on the air conditioning system and lower building operational energy consumption. Additionally, a good indoor thermal and humidity environment can prevent the growth of mold caused by excessive humidity, reduce the likelihood of respiratory diseases among occupants, and improve health performance.

Natural lighting plays an important role in indoor environmental quality and building energy consumption. Under the premise of meeting lighting requirements, reducing the use of artificial lighting can effectively lower the operational energy consumption of the building. Well-designed windows can improve the uniformity of indoor illumination, ensuring that main activity spaces receive sufficient natural light while avoiding glare. Optimizing window parameters can ensure that the indoor lighting environment remains within a healthy range for most of the year. This can reduce the need for artificial lighting and lower electricity consumption in the building (16, 17). Additionally, the use of natural light can improve occupants’ physiological rhythms, and enhance their quality of life and health levels.

To sum up, many factors affect the energy consumption and health performance of residential buildings in hot-summer and cold-winter regions. These factors include building envelope, heating and cooling systems, indoor environmental control, and natural lighting. Among numerous factors, this work focuses on the analysis and optimization research of the thermal and optical properties of window glass to highlight its crucial role in enhancing building energy efficiency and creating a healthy indoor environment.

### 2.2 Analysis of types and technical principles of high-performance glass

High-performance glass, as one of the essential materials in modern buildings, encompasses various types and technical characteristics, primarily including Low-E glass, U-shaped glass, photovoltaic glass, and vacuum glass (18–20). These glass materials are optimized during design and manufacturing processes to meet different functional needs, enhancing their performance in energy savings, sound insulation, and improving indoor environmental quality.

**Figure 2:**
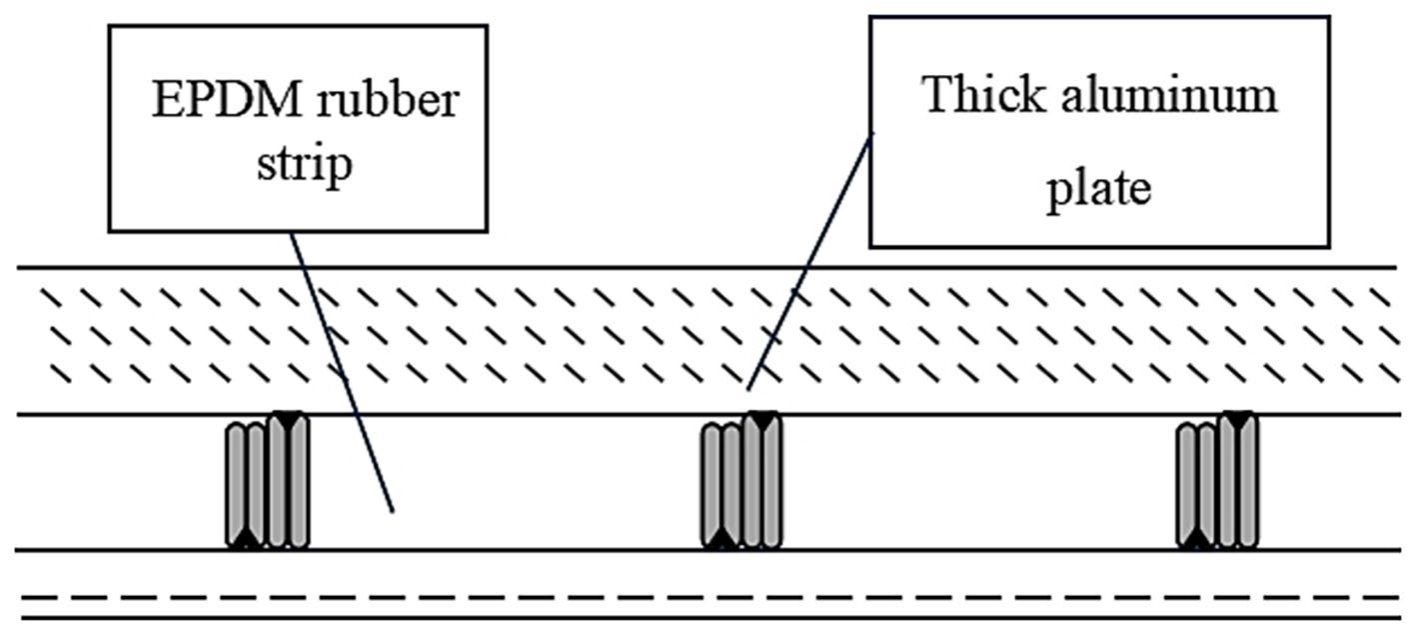
Double-row combination form of U-shaped glass.

Low-E glass effectively reduces radiative heat loss by coating a Low-E film on the glass surface, thereby improving the building’s insulation performance. It can reflect indoor heat, reducing energy loss in winter, while blocking heat entry in summer, achieving year-round energy-saving effects. This type of glass can also filter UV rays, protecting indoor furniture and decorations from UV damage and extending their lifespan. Additionally, Low-E glass has good light transmittance, ensuring natural lighting indoors and enhancing the comfort of living and working environments. Photovoltaic glass integrates solar cell technology, converting solar energy into electrical power. It not only serves as a decorative material for building facades but also provides part of the building’s power needs, achieving self-sufficiency and sustainable development. Photovoltaic glass, when integrated into buildings, not only reduces reliance on traditional energy sources but also decreases the building’s carbon footprint, promoting the development of green buildings. With ongoing advancements in photovoltaic technology, the electricity generation efficiency and cost-effectiveness of photovoltaic glass are continually improving, making large-scale applications in buildings increasingly feasible. Vacuum glass forms a vacuum layer between two glass panels, effectively isolates heat conduction and convection, and significantly enhances the insulation performance of the glass. It has an extremely low thermal conductivity coefficient, making it one of the best insulating building glass materials available. Due to its vacuum layer which eliminates thermal losses caused by air convection, vacuum glass effectively maintains indoor warmth during winter and isolates outdoor heat during summer, significantly reducing building energy consumption. Additionally, vacuum glass offers excellent sound insulation, effectively blocking external noise and enhancing the quietness of indoor environments. U-shaped glass features an insulating layer in the center of the glass, effectively isolates external heat and noise, and enhances the building’s soundproofing effects, as shown in Figure 2. This structural design not only reduces heat transfer but also lowers noise pollution inside the building, improving the living quality of occupants. U-shaped glass is widely used in the exterior wall and window designs of buildings, especially in places requiring high sound insulation and thermal insulation performance, such as airports, hospitals, and upscale residential areas.

Table 1 displays the comparison of the advantages and disadvantages of various high-performance glass types (21–23).

**Table 1:** Comparison of various high-performance glass types.

High-performance glass types	Application advantages	Application disadvantages	Suitable building types
Low-E Glass	Excellent thermal insulation and light transmission, significant energy-saving effect.	Requires regular cleaning and maintenance of the coating layer.	Commercial office buildings, and residential buildings
U-Shaped Glass	High strength, easy construction, significant energy-saving effect.	Higher initial cost, and high design requirements for glass.	Large public buildings, and stadiums
Photovoltaic Glass	Provides green energy, and reduces indoor lighting costs.	High initial investment; technology maturity and market application need further improvement.	Commercial office buildings, and residential buildings
Vacuum Glass	Best insulation performance, significant energy-saving effect.	High manufacturing cost, and high installation technology requirements.	High-end residential buildings, and research institutions.

The application of high-performance glass in buildings not only significantly enhances the energy efficiency of the structures but also effectively improves indoor environmental quality. For example, Low-E glass reduces energy costs by controlling heat transfer, thereby decreasing heating consumption in winter and air conditioning consumption in summer. U-shaped glass and vacuum glass, with their excellent thermal insulation properties, reduce the transfer of temperatures between the interior and exterior of the building, thus improving overall insulation and enhancing the comfort of the living environment. The application of photovoltaic glass enables buildings to harness renewable energy, reduces dependence on traditional electricity sources, and enhances the building’s energy self-sufficiency and environmental friendliness.

Overall, the use of high-performance glass can effectively reduce energy consumption and carbon emissions in buildings, while also increasing the building’s value and improving the quality of life for its occupants. A deep understanding of the different types of high-performance glass and their technical principles can better guide their rational application in buildings, driving the construction industry toward a green, low-carbon, and sustainable direction. This not only meets the current global demand for environmental protection but also provides crucial technological support for improving future urban development and human living environments.

### 2.3 Multi-objective optimization model based on GA

#### 2.3.1 GA-based optimization method

To solve the complex non-linear trade-off problem between building glass performance parameters and multiple objectives (energy consumption, carbon emissions, and health performance), GA is selected as the core optimization tool. GA’s strong global search capability and multi-objective optimization capability can effectively balance the complex trade-offs of building glass performance among energy consumption, carbon emissions, and health performance, improving optimization efficiency and the scientific nature of results. In addition, GA can directly generate a set of well-distributed Pareto optimal solution sets, thereby providing decision-makers with multiple performance trade-off schemes and supporting final design decisions. GA is a random optimization algorithm that simulates the natural selection process. It continuously applies operations such as selection, crossover, and mutation in the solution space, ultimately approaching the global optimal solution (24, 25). Here, GA is mainly employed to optimize the performance parameters of building glass while considering multiple optimization objectives, such as building operational energy consumption, lifecycle carbon emissions, and indoor health performance. The core of GA is the fitness function, which determines the “quality” of each individual in the current population, essentially evaluating how well a solution performs (26). Here, building lifecycle carbon emissions, operational energy consumption, and indoor health performance improvements are set as optimization objectives. Therefore, a comprehensive fitness function needs to be designed to unify these objectives and transform them into either minimization or maximization forms. Specifically, the fitness function in this work is:


$$\max f(x)=\{\begin{array}{c} \min F_{\mathrm{car}}(x)\\ \min F_{\mathrm{ene}}(x)\\ \max F_{\mathrm{hea}}(x) \end{array}$$ (1)

**Figure 3:**
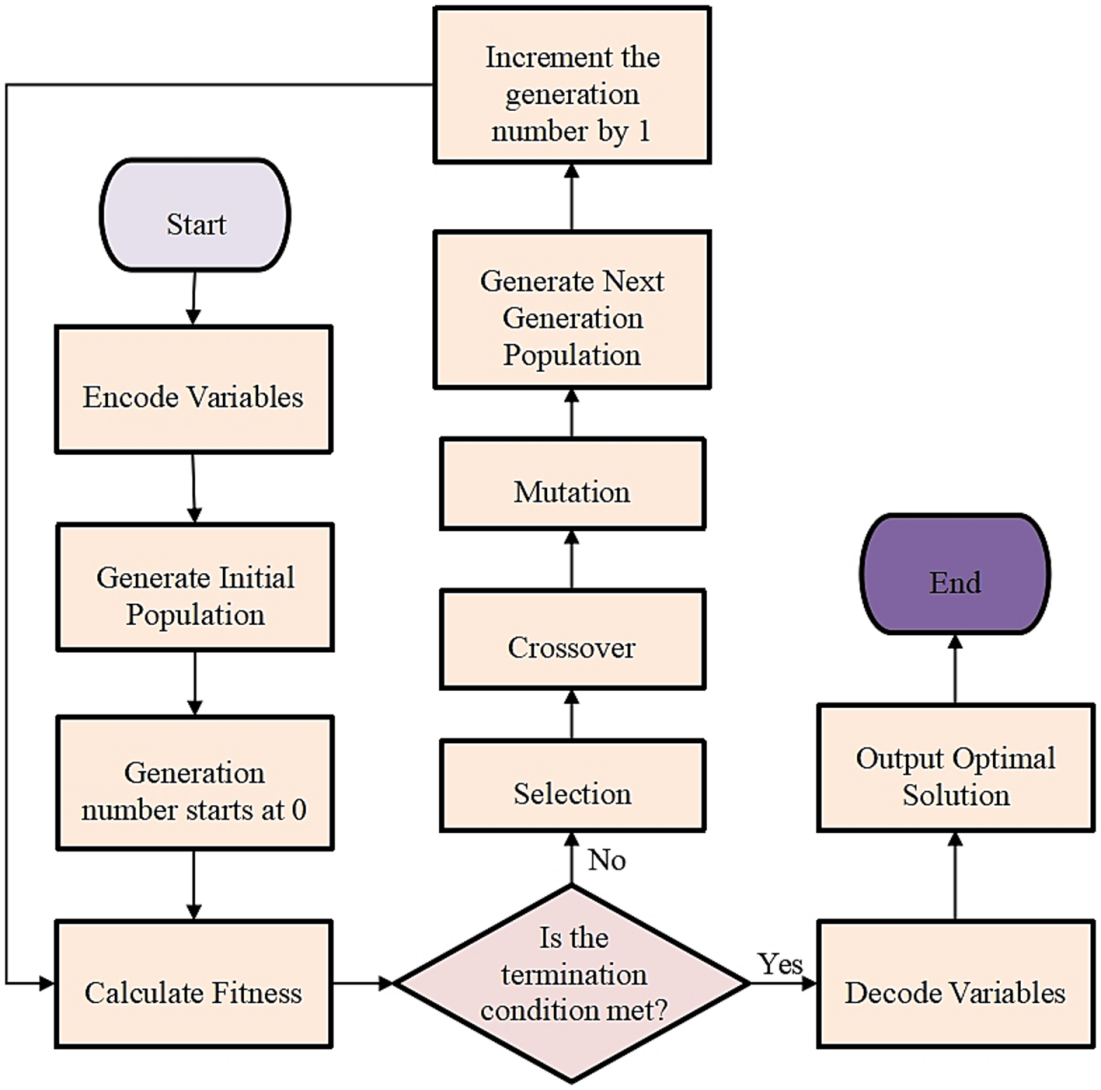
GA-based optimization process.

In this context, $F_{\mathrm{car}}(x)$ represents the calculation function for the building’s lifecycle carbon emissions, which is an objective to be minimized, that is, the lower the carbon emission is, the better it is. $F_{\mathrm{ene}}(x)$ represents the calculation function for the building’s operational energy consumption, which is also a minimization objective. Optimizing the glass performance can reduce the building’s energy consumption. $F_{\mathrm{hea}}(x)$ represents the calculation function for the indoor health performance of the building, with health performance being a positive objective to maximize, that is, the healthier the indoor environment is, the better it is. Through the iterative process of the GA, the selection, crossover, and mutation operations continuously adjust the glass parameters of each individual, aiming to minimize carbon emissions and energy consumption while maximizing indoor health performance. During the optimization process, the GA evaluates each solution based on the above fitness functions, progressively seeking the optimal combination of glass performance parameters. Figure 3 illustrates this process.

Relevant studies on the application of GA in building performance optimization have been referred to. These studies indicate that for moderately complex building optimization problems, a population size of 50–100 and 100–200 evolution generations typically achieve effective convergence. This parameter range maintains a good balance between computational efficiency and solution quality. Meanwhile, in preliminary tests, when the population size is 50 and the number of iterations is 100, the algorithm achieves a good balance between convergence speed and solution stability. Besides, the optimization results have high accuracy and ensure computational efficiency. Thus, the population size is set to 50, the number of generations is 100, the crossover rate is 0.9, and the mutation rate is 0.01.

#### 2.3.2 Indoor health performance objectives for residential buildings

In residential buildings, optimizing indoor health performance is a key factor in affecting the health and comfort of occupants. To ensure that the building environment provides a healthy and comfortable living space for residents, this work primarily focuses on two important indoor health performance objectives: thermal-humidity environment and lighting environment. The thermal-humidity environment refers to the impact of the combination of indoor air temperature and relative humidity on the health and comfort of residents. According to the international standard American Society of Heating, Refrigerating and Air-Conditioning Engineers (ASHRAE), indoor temperatures should be maintained between 18 °C and 26 °C; moreover, the relative humidity should remain within 30 to 70% to ensure thermal comfort and avoid the negative health effects of too high or too low humidity levels. Here, the health performance of the thermal-humidity environment is evaluated by calculating the percentage of hours that the indoor thermal-humidity environment meets health requirements, and this is used as one of the optimization objectives for GA. To quantify this indicator, the Predicted Mean Vote (PMV) standard is used as the evaluation criterion, and indoor thermal comfort is assessed hourly based on the ASHRAE standards. Then, the percentage of time during the year when the thermal-humidity environment meets health requirements is calculated. The lighting environment’s health performance is also critical to the health and quality of life of occupants. According to the healthy building evaluation standards, interior spaces (such as bedrooms, studies, and living rooms) must ensure adequate natural lighting, and indoor illuminance should be maintained between 300 lx and 500 lx. The illuminance uniformity should be not less than 0.40, and the daylight glare probability (DGP) should not exceed 0.45. To quantify the lighting environment’s health performance, the percentage of time that each major interior space meets the required illuminance, uniformity, and glare index under natural lighting conditions is calculated, and the total percentage of healthy lighting hours for the year is derived.

By combining the health performance objectives of the thermal-humidity and lighting environments, the goal is to maximize the percentage of healthy indoor hours throughout the year by optimizing the glass performance parameters of the windows. To this end, this work uses the comprehensive healthy hours percentage (H) as the objective function for optimization, as shown in the following equation:


$$H=\alpha_{1}\cdot H_{\text{the}}+\alpha_{2}\cdot H_{\mathrm{lig}}$$ (2)

$$H_{\mathrm{lig}}=\frac{H_{E}+H_{U}+H_{\mathrm{DGP}}}{3}$$ (3)

Here, $H_{\text{the}}$ represents the percentage of healthy hours for the thermal-humidity environment, and $H_{\mathrm{lig}}$ represents the percentage of healthy hours for the lighting environment. $\alpha_{1}$ and $\alpha_{2}$ are the weights for the thermal-humidity and lighting environments, respectively, with values of 7/12 and 5/12. $H_{E}$ , $H_{U}$ , and $H_{\mathrm{DGP}}$ represent the annual indoor illuminance, illuminance uniformity, and DGP healthy hours percentage, indicating the proportion of time during the year when indoor illuminance, illuminance uniformity, and DGP meet health requirements. The core of this optimization objective is to maximize the percentage of healthy hours for both indoor thermal-humidity and lighting environments by optimizing glass performance, thereby enhancing the health and comfort of the occupants.

#### 2.3.3 Operational energy consumption objective

In building design and optimization, operational energy consumption is a key indicator for assessing building energy efficiency and environmental impact. The energy consumption of a building mainly comes from the air conditioning, heating, and lighting systems, and the energy usage of these systems is closely related to indoor thermal-humidity conditions and the quality of the lighting environment. Here, the operational energy consumption objective of buildings focuses on the electricity consumption of air conditioning, heating, and artificial lighting systems, aiming to reduce overall energy consumption through the optimization of glass performance and building design. The thermal-humidity environment indoors has a significant impact on the energy consumption of the air conditioning and heating systems. To maintain a comfortable indoor thermal-humidity environment, the air conditioning and heating systems need to adjust according to external climate changes, especially during extreme seasonal conditions, where system load is high, leading to increased energy consumption. Therefore, the energy consumption of the air conditioning and heating system is considered one of the key indicators for evaluating the building’s operational energy efficiency in the optimization model. When natural lighting is insufficient, the artificial lighting system is activated to ensure that indoor illuminance meets health requirements. According to worldwide relevant building design standards, the indoor health illuminance requirement for residential buildings is 300–500 lx. When lighting conditions fall below 300 lx, the artificial lighting system will turn on to supplement the lighting. Thus, the energy consumption of the lighting system is also an important component of the building’s total energy consumption. To quantify the building’s operational energy consumption, the total electricity consumption per unit area is set as the optimization objective, as shown in the following equation:


$$E=E_{\mathrm{kt}}+E_{\mathrm{zm}}$$ (4)

Here, E represents the total operational energy consumption of the building, indicating the total energy consumption per unit area of the building over the course of 1 year. $E_{\mathrm{kt}}$ is the energy consumption of the air conditioning and heating system, and $E_{\mathrm{zm}}$ is the energy consumption of the lighting system. The units for all three energy consumptions are kilowatt-hours per square meter per year [kWh/(m^2^·a)]. The optimization objective is to enhance the quality of the indoor thermal-humidity environment and lighting environment by improving the window glass performance. This is intended to reduce the energy consumption of the air conditioning, heating, and lighting systems, and ultimately lower the building’s overall operational energy consumption. In this process, the optimization plan must not only consider energy-saving effects but also balance the comfort and health of the indoor environment.

#### 2.3.4 Carbon emission objective

The carbon emissions over the entire life cycle of a building are one of the key indicators for assessing its environmental impact. This work analyzes the carbon emissions throughout the building’s life cycle and explores the impact of different window technology parameters on carbon emissions. The carbon emissions over the entire life cycle of a building mainly consist of three stages: the material production and transportation stage, the construction and demolition stage, and the operational stage.

During the building’s material production and transportation stage, the production and transportation of glass involve multiple steps, each generating a certain amount of carbon emissions. It should be noted that this work mainly focuses on the environmental dimension in life cycle assessment. As a result, it focuses on quantifying carbon emissions at each stage as an environmental midpoint indicator. The embodied carbon generated in the material production stage has been taken into account in the calculation, which, to a certain extent, reflects the environmental cost brought by the initial investment in high-performance glass. Although economic cost (i.e., initial investment) is an important factor in engineering decision-making, this work focuses on establishing an optimization model for environmental performance. Therefore, economic parameters are not included in the objective function. The carbon emissions from the glass production and transportation stage are calculated using the following equation:


$$C_{\mathrm{SY}}=C_{\mathrm{sc}}+C_{\mathrm{ys}}$$ (5)

$C_{\mathrm{SY}}$ represents the carbon emissions during the glass production and transportation phase, measured in kilograms of carbon dioxide equivalent per square meter (kgCO_2_e/m^2^); $C_{\mathrm{sc}}$ refers to the carbon emissions during the glass production phase, and $C_{\mathrm{ys}}$ represents the carbon emissions during the glass transportation phase, measured in kgCO_2_e.

The carbon emissions from the construction and demolition phases typically account for a smaller portion of the building’s total life-cycle carbon emissions, so this work assumes them to be negligible (set to 0).

The operational phase of the building mainly includes the power consumption of the heating, ventilation, and air conditioning (HVAC) system and the lighting system. During this phase, electricity is the primary energy source, and the building’s carbon emissions are mainly related to the carbon emission factor of the power grid and the building’s energy consumption level. The carbon emissions during the operational phase of the building are calculated using the following equation:


$$C_{\mathrm{YX}}=\frac{(E_{\mathrm{kt}}+E_{\mathrm{zm}})\times E_{\mathrm{Fe}}\times y}{A}$$ (6)

$C_{\mathrm{YX}}$ represents the carbon emissions during the building’s operational phase, measured in kgCO₂e/m^2^; $E_{\mathrm{kt}}$ is the annual electricity consumption of the building’s HVAC system; $E_{\mathrm{zm}}$ is the annual electricity consumption of the building’s lighting system, measured in kilowatt-hours per year (kWh/a); $E_{\mathrm{Fe}}$ is the average CO₂ emission factor of the regional power grid in China; y is the building’s design life, measured in years; and A is the building’s floor area, measured in m^2^.

The carbon emission optimization objective is to reduce the unit area carbon emissions of the building throughout its entire life cycle by optimizing the window parameters. While achieving the optimal thermal-humidity environment and lighting environment for the building, this also further reduces the building’s carbon footprint. Reasonable design and optimization of window technical parameters can reduce the building’s energy consumption and decrease carbon emissions during the material production, transportation, and operational phases. This can achieve the goal of green and low-carbon buildings while maintaining the building’s functionality and comfort.

#### 2.3.5 Feasibility constraints of glass performance parameters

During GA-based multi-objective optimization, treating glass thermal and optical parameters—such as K-value, SHGC, and VT—as entirely independent variables may produce theoretically optimal configurations. However, such solutions could prove physically unattainable or commercially unavailable in practice. Explicit feasibility constraints are introduced into the optimization model to ensure the physical rationality and engineering practicality of optimization results. These constraints are mainly based on the physical principles of high-performance glass and commercial product databases.

Specifically, there is an inherent physical correlation between SHGC and VT, as both are jointly affected by the coating properties of the glass surface, the number of layers, and the intermediate gas medium. Generally, for glass with similar coating types, higher VT is usually accompanied by higher SHGC. To quantify this relationship, regression analysis is performed on the SHGC and VT of 1,075 glass samples from the database. The analysis references both the ASHRAE Handbook—Fundamentals and product databases from major glass manufacturers such as Saint-Gobain and AGC. This leads to the establishment of a feasible region boundary constraint as shown in Equation 7:


0.8×VT−0.15≤SHGC≤1.2×VT−0.1 (7)

This constraint ensures that when the optimization algorithm searches the solution space, it automatically excludes design schemes where the combination of SHGC and VT violates basic physical laws. In addition, the K-value of glass is closely related to the number and thickness of its insulating layers, as well as the filled gas (such as air and argon). A lower (better) K-value usually requires a more complex insulating layer structure or the use of low-conductivity gas, which may have a negative impact on VT to a certain extent. In the work, the generation of the initial population of optimization variables and iterative mutation operations is strictly restricted within the scope of the commercial glass product parameter database. This means that every combination of glass performance parameters generated by the algorithm during the “selection,” “crossover,” and “mutation” operations corresponds to an actually existing glass product or one that can be manufactured using existing technologies.

By introducing the above-mentioned physical correlation constraints and commercial product database screening, the multi-objective optimization model can effectively limit the search space to a “feasible solution” region. This ensures that all Pareto optimal solutions have engineering feasibility and enhances the practical value and guiding significance of the optimization results.

### 2.4 Case selection and data collection

**Figure 4:**
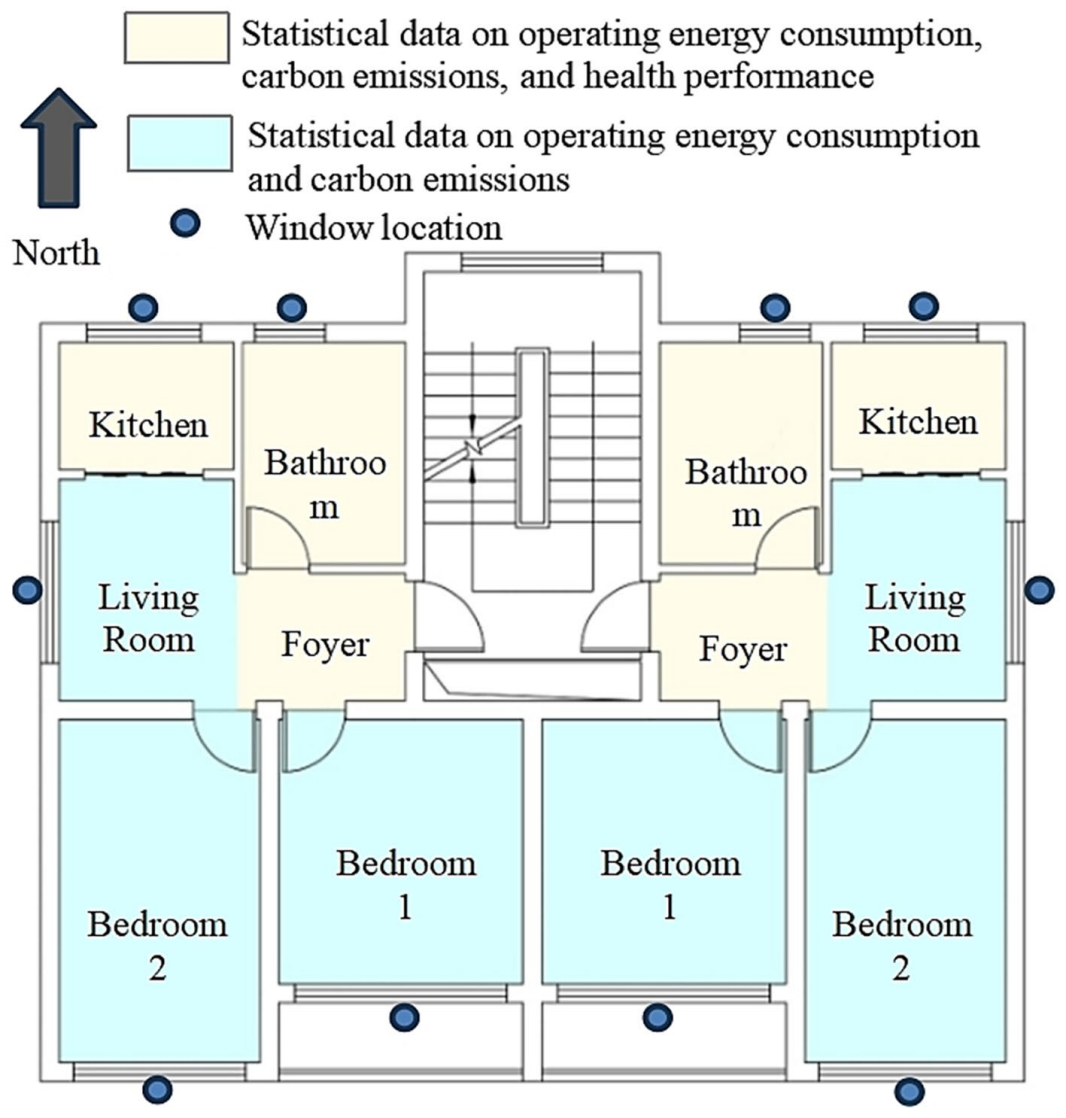
Standard floor plan layout.

This work selects a typical residential building located in the hot-summer and cold-winter regions of Hangzhou as the research object. A three-dimensional model of the building is created in Rhino software for subsequent building performance simulation and optimization analysis. The building has six floors, with a form factor of 0.39 for the above-ground portion. It does not have an elevator, and the overall layout is compact with reasonable functional zoning. The standard floor layout adopts a mirrored design of two east–west units, with each floor covering an area of 120 m^2^, ensuring good residential comfort and space utilization. The building’s interior space includes two bedrooms, a living room, a kitchen, and a bathroom, covering the main functional areas of a typical residence. The building’s exterior windows are configured with double-glazed insulated glass consisting of 6 mm Low-E glass, 12 mm air gap, and 6 mm transparent glass. Figure 4 shows the floor plan of the building.

For accurate building performance simulation, detailed indoor environment parameters are set in the work as the basis for energy simulation and health performance evaluation. Indoor thermal disturbance settings include occupants, lighting, and equipment. The occupant schedule is set according to typical residential routines: 2 people are set for weekday nights (23:00 to 7:00 the next day), 1 for weekday days (7:00 to 18:00), and 2 for weekday evenings (18:00 to 23:00); on weekends, 2 people are set to be present for most of the day. The occupant’s metabolic rate takes the typical value of 1.0 met under resting conditions. Clothing thermal resistance is adjusted according to the season: 0.5 clo in summer and 1.0 clo in winter, to reflect actual dressing habits. The set temperature for heating is 20 °C, and the set temperature for cooling is 26 °C; the system operation time is synchronized with the occupant presence time. The lighting power density is set to 5 W/m^2^, and the equipment power density is set to 4 W/m^2^; their operation schedules are related to occupant activities.

The collected data includes the window unit, specifically the glass, with parameters such as the thermal transmittance (K-value), SHGC, and VT, which directly relate to the building’s energy efficiency and further influence the building’s carbon emissions. The thermal transmittance of the glass determines the efficiency of heat transfer through the window. A higher K-value means that the window will result in more heat loss during winter, increasing the energy consumption and carbon emissions of the HVAC system. SHGC, on the other hand, directly affects the window’s absorption of solar radiation heat, thereby influencing the indoor thermal environment. A higher SHGC can lead to overheating, increasing the load on the air conditioning system. In the simulation, the calculation of mean radiant temperature fully considers the temperature of each indoor surface. Among them, the inner surface temperature of the glass is obtained through dynamic heat balance calculation based on its K-value, the indoor-outdoor temperature difference, and solar radiation heat gain (comprehensively considering SHGC and incident angle correction). This ensures the accuracy of the radiant temperature component in the PMV evaluation index.

To ensure the scientific accuracy and reliability of the data, a residential building glass parameter database is established. This database is based on the ASHRAE Handbook Fundamentals, the Zhejiang Province Residential Building Energy Efficiency Design Standard DB33/1015–2015, and related engineering practices. It includes 1,075 different types of glass, with each entry containing four key parameters: average thermal transmittance, SHGC, VT, and embodied carbon emissions, providing detailed data support for subsequent research. From the perspective of reducing the types of components in the actual project, the simulation sets the windows on the same facade to be identical. It means the performance parameters of the glass in each window component on the same facade are the same. In the simulation, glass performance parameters are input based on the database.

## 3 Results and discussion

### 3.1 Analysis of the results for glass performance parameter optimization

Based on the building performance optimization with meteorological parameters from Hangzhou, the initial building performance parameters are obtained from the simulation, as shown in Table 2.

**Table 2:** Initial building performance parameters.

Parameter name	Result	Sub-parameter name	Result
Operating Energy Consumption [kWh/(m^2^·a)]	50.95	–	–
Carbon Emissions (kgCO₂e/m^2^)	2622.93	–	–
Indoor Health Time Proportion (%)	34.46	PMV	32.87%
		Illuminance	11.76%
		Illuminance Uniformity	50.23%
		Glare	48.09%

**Figure 5:**
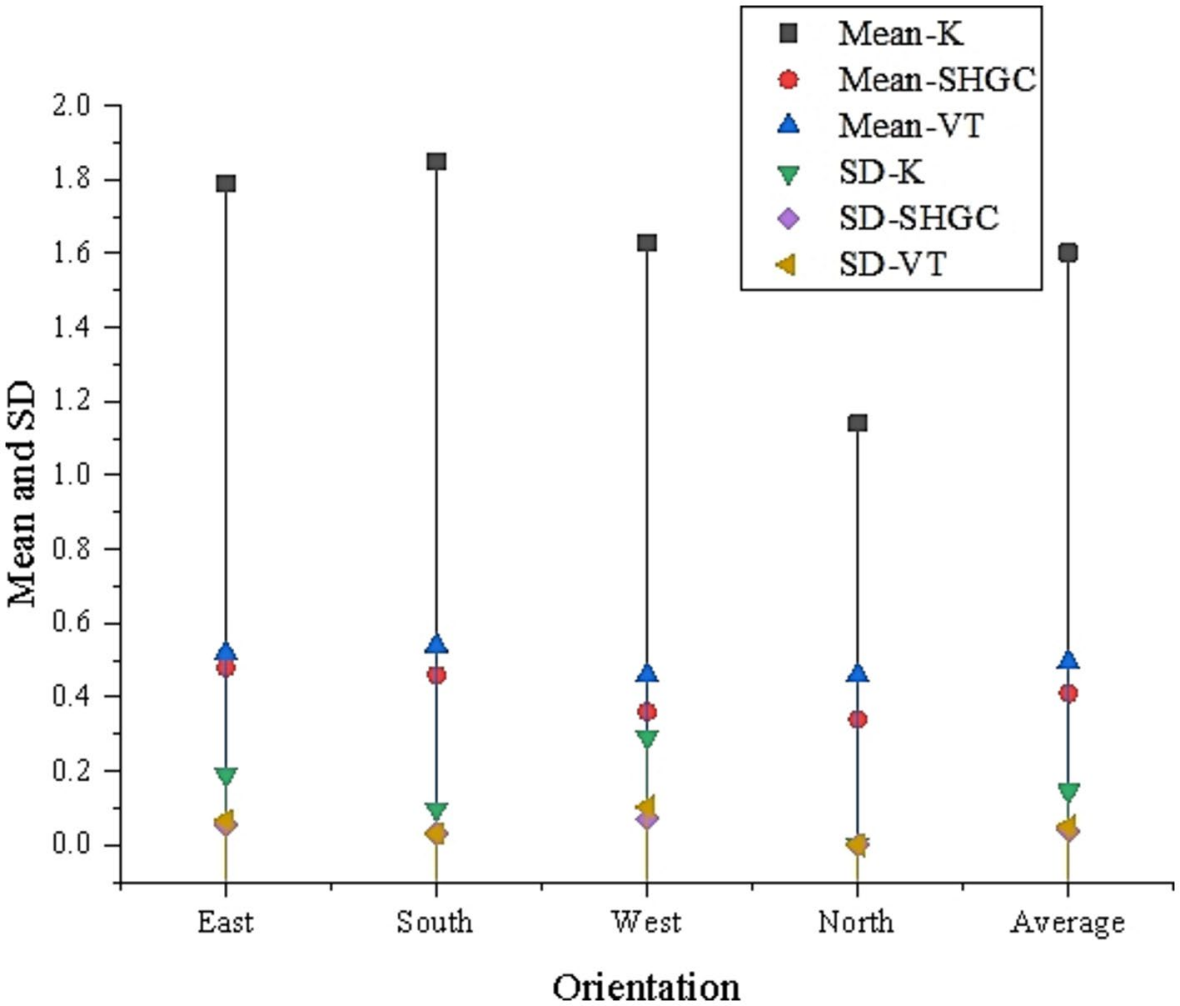
Optimization results of glass performance parameters.

After the GA calculation, a total of 55 Pareto optimal solutions are obtained. Figure 5 shows the mean and standard deviation (SD) of the solutions to glass performance parameter optimization for different orientations.

Figure 5 reveals that there are certain differences in the glass thermal transmittance, SHGC, and VT for the four orientations: East, South, West, and North. The average thermal transmittance for the East-facing windows is 1.79, SHGC is 0.48, and VT is 0.52, with standard deviations of 0.19, 0.054, and 0.066, respectively, indicating significant performance fluctuations for the East-facing windows. The glass performance for the South-facing windows is relatively stable, with an average thermal transmittance of 1.85, SHGC of 0.46, and VT of 0.54. The optimization results for the West-facing windows show a lower thermal transmittance (1.63) and SHGC (0.36). The optimization results for the North-facing windows show the lowest thermal transmittance, at 1.14. Overall, the optimization results for South-facing windows are more stable and perform better, while the optimization results for East and West-facing windows fluctuate more. The optimization parameters for the North-facing windows are the least favorable, with a significantly lower thermal transmittance, which impacts the building’s energy efficiency.

**Figure 6:**
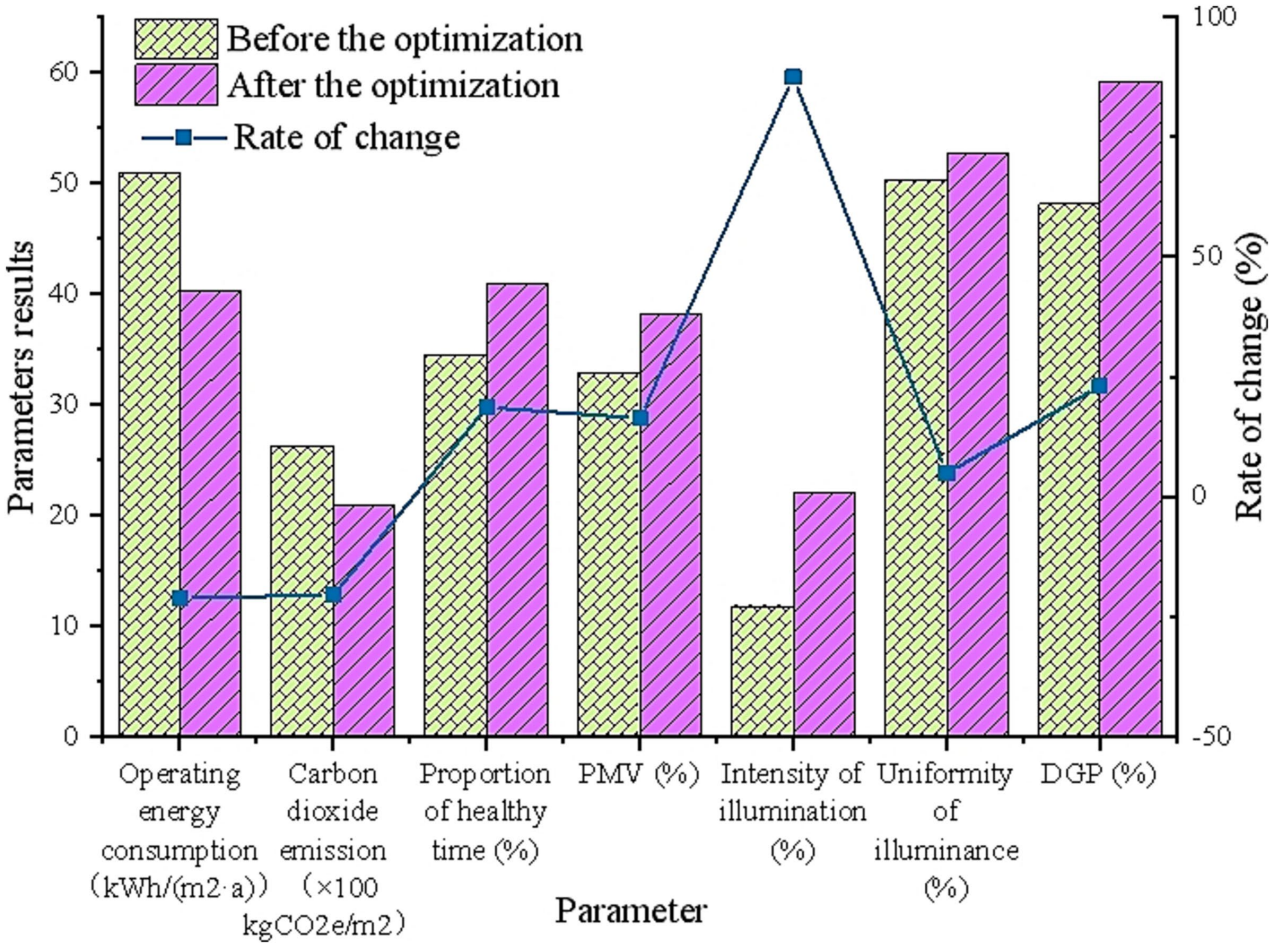
Examples of high-performance glass applications.

Figure 6 shows the mean performance parameters of the window glass in the Pareto optimal solutions.

According to Figure 6, the glass performance parameters for the four orientations show significant differences, reflecting the impact of windows facing different directions on building energy efficiency and indoor environment. In terms of thermal transmittance, the optimization result for South-facing windows is the best, with an average value of 1.842, noticeably higher than the other orientations. The average thermal transmittance for East-facing windows is 1.779, slightly lower than South-facing windows, but still higher than West and North-facing windows. This suggests that South-facing windows offer better thermal performance, reducing heat loss during winter. Regarding SHGC, South-facing windows have a value of 0.459, slightly higher than the other orientations, indicating that South-facing windows can more effectively utilize solar radiation heat to improve winter energy efficiency. On the other hand, North-facing windows are the least favorable for heat absorption. For VT, South-facing windows have a value of 0.538, which is relatively high, meaning they provide better natural daylighting. Overall, South-facing windows perform well in terms of thermal performance, solar heat gain, and natural daylighting, making them suitable for optimization design.

In summary, for residential buildings in summer-hot and winter-cold regions, optimizing glass performance parameters is not only related to the building’s energy efficiency and carbon emissions but also directly impacts the indoor health environment. From the GA optimization results, it is clear that South-facing windows stand out in the overall optimization. Their higher SHGC and VT contribute to improving winter energy efficiency and natural daylighting, and effectively reducing heating energy consumption and carbon emissions. Additionally, South-facing windows perform better in terms of thermal-humidity and lighting environments, enhancing indoor comfort and health. Therefore, for building designs in summer-hot and winter-cold regions, when optimizing the glass performance, it is recommended to prioritize the optimization of South-facing windows. A higher thermal transmittance and appropriate SHGC can be adopted to improve building energy efficiency and reduce carbon emissions. For East and West-facing windows, appropriate adjustments should be made based on specific conditions, especially in areas with higher heat load, where SHGC can be reduced to mitigate the risk of overheating in summer. Furthermore, the lower thermal performance and light conditions of North-facing windows indicate that measures, such as optimizing window materials or adding daylighting devices, may be required to compensate for their shortcomings, ensuring the building’s energy efficiency and indoor health throughout the year. In summary, when optimizing glass performance parameters, in addition to reducing energy consumption and carbon emissions, the improvement of the indoor health environment should be fully considered. In particular, thermal-humidity and lighting environments should be considered to achieve a comprehensive, multi-objective optimization.

### 3.2 Comparison of building operational performance before and after optimization

Among all the Pareto optimal solutions, five glass optimization schemes that best align with the actual project in Hangzhou based on a typical building were selected, as shown in Table 3.

**Table 3:** Typical residential glass optimization schemes for Hangzhou region.

Scheme	Scheme	South	West	North
Scheme 1	3 + 6A + 3Low-E	3 + 13Ar + 3Low-E	6 + 6Ar + 6Low-E	6Low-E+13Ar + 6 + 13Ar + 6
Scheme 2	6 + 13Ar + 6Low-E	3 + 13Ar + 3Low-E	6 + 6Ar + 6Low-E	6Low-E+13Ar + 6 + 13Ar + 6
Scheme 3	3 + 6A + 3Low-E	6 + 13Ar + 6Low-E	6 + 6Ar + 6Low-E	6Low-E+13Ar + 6 + 13Ar + 6
Scheme 4	3 + 13Ar + 3Low-E	6 + 13Ar + 6Low-E	6 + 6Ar + 6Low-E	6Low-E+13Ar + 6 + 13Ar + 6
Scheme 5	3 + 13Ar + 3Low-E	3 + 6A + 3Low-E	6 + 6Ar + 6Low-E	6Low-E+13Ar + 6 + 13Ar + 6

A denotes the air layer, and Ar denotes the argon gas layer. All numbers omit the unit “mm.” For example, “3 + 6A + 3Low-E” indicates the window glass composition of 3 mm regular glass + 6 mm air layer + 3 mm Low-E glass.

**Figure 7:**
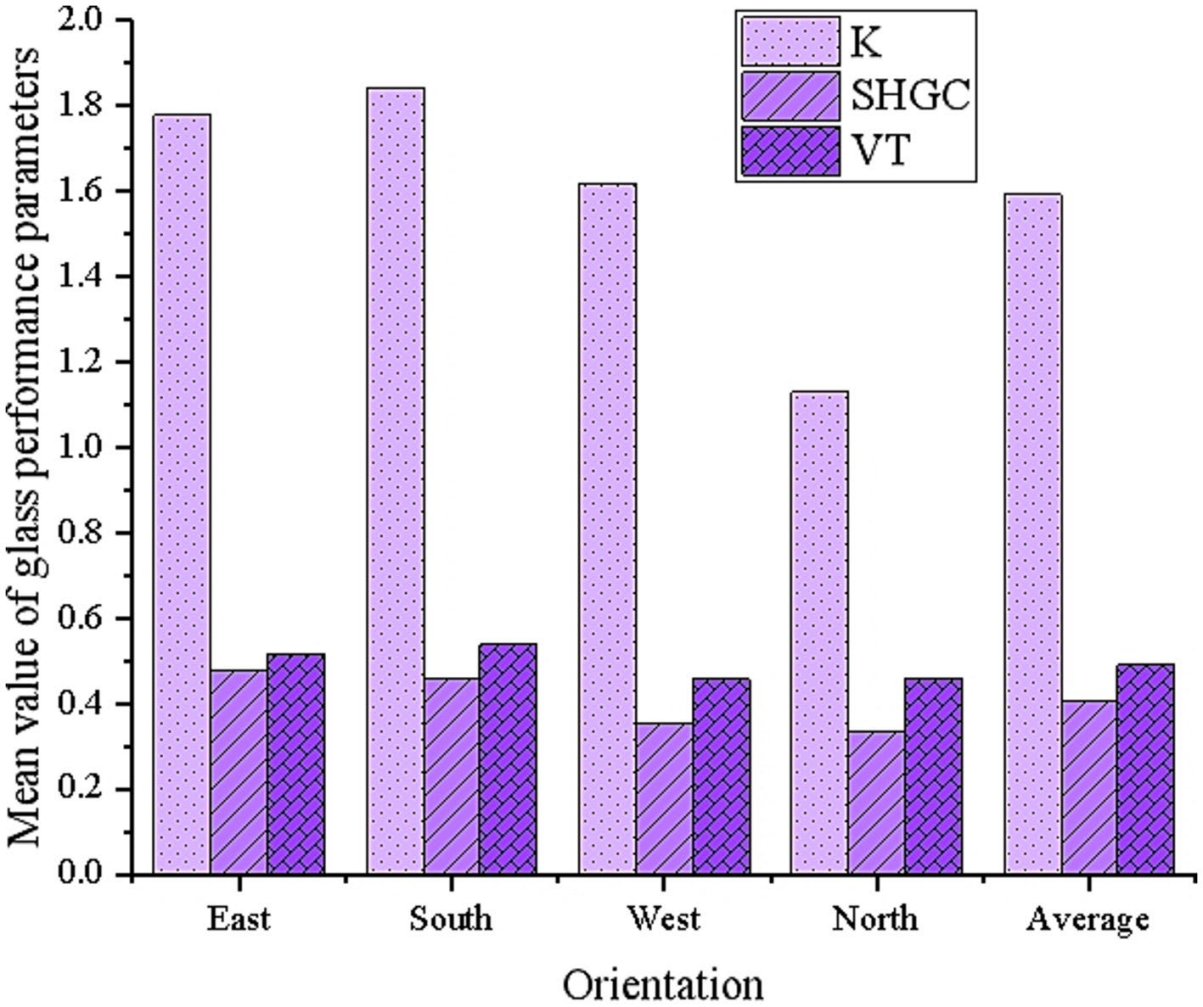
Comparison between traditional glass and high-performance glass.

Figure 7 shows the results of operational energy consumption, carbon emissions, and the proportion of healthy time before and after using the optimization schemes. The post-optimization results are presented as averages.

Figure 7 shows that the optimized window glass performance significantly improves the building’s operational energy efficiency, reduces carbon emissions, and enhances the indoor health environment. First, the optimized building’s energy consumption decreases from 50.95 kWh/(m^2^·a) to 40.26 kWh/(m^2^·a), reducing energy consumption by 20.98%, indicating that the optimization scheme effectively improves the building’s energy efficiency. Besides, carbon emissions are also significantly reduced, from 2,623 kgCO₂e/m^2^ to 2083 kgCO₂e/m^2^, a decrease of 20.57%, further proving the positive environmental impact of the optimization scheme. In terms of health performance, the proportion of healthy indoor time increases from 34.46 to 40.9%, an increase of 18.69%. This suggests that the optimized glass parameters significantly improve the thermal-humidity and light environments, and enhance the comfort of the occupants. Specifically, the PMV ratio increases by 16.31%, and the uniformity of illuminance also improves, particularly in the enhancements to illuminance intensity and DGP, demonstrating the optimization scheme’s effectiveness in enhancing natural daylighting and reducing glare.

In summary, the window glass performance optimized through GA shows significant improvements in multiple aspects. The optimization scheme not only effectively improves the building’s operational energy efficiency and reduces carbon emissions but also significantly enhances the indoor health environment, especially in optimizing the thermal-humidity and light conditions. The optimized glass design improves indoor thermal comfort, illuminance uniformity, illuminance intensity, and DGP, thereby enhancing the comfort and health experience of the occupants. Furthermore, the optimization scheme adjusts windows for different orientations. In particular, South-facing windows fully utilize solar radiation heat and natural daylighting, which effectively reduces the building’s air conditioning and heating energy consumption, thus further lowering carbon emissions. In building design for summer-hot and winter-cold regions, prioritizing the optimization of South-facing windows maximizes the building’s energy efficiency and indoor comfort. For East, South, and West-facing windows, adjustments should be made based on varying heat load demands; optimized designs can mitigate the risk of overheating in summer and further enhance the building’s overall energy efficiency. Overall, the proposed glass performance optimization scheme provides strong support for multi-objective optimization of building energy efficiency, carbon emissions, and indoor health performance, offering practical guidance for low-carbon building design.

## 4 Discussion

Through GA optimization, this work obtains glass performance parameter combinations that balance energy consumption, carbon emissions, and health performance. The optimization results show that windows with different orientations have systematic differences in their requirements for glass performance parameters, which reflects the different climatic driving forces faced by external building windows. Compared with the study by Wang et al. (27), this work also adopts a multi-objective optimization method. However, their study mainly focused on improving light transmittance through the thin-film interference principle of multi-layer thermochromic smart windows to balance energy consumption, anti-glare, and daylighting. In contrast, this work uses GA to integrate energy consumption, carbon emissions, and health performance simultaneously within a broader scope, proposes a comprehensive optimization framework. Meanwhile, targeted optimization is conducted for windows with different orientations—demonstrating the method’s universality and adaptability. Shaik et al. (28) addressed the energy consumption issue of glass buildings by developing three double-glazed systems with organic phase change materials (21/30/35 °C). This system could save operating costs while maintaining sufficient natural daylighting. However, this work optimizes material parameters and comprehensively considers building operating energy consumption and life-cycle carbon emissions; it also introduces health performance as an optimization objective, forming a more comprehensive multi-objective optimization system. Volf et al. (29) conducted a field experiment in 72 apartments to compare the impact of double-layer high-light-transmittance glass and triple-layer low-energy glass on residents’ health. They found that although high-light-transmittance glass saved 11.0% energy and was rich in circadian rhythm spectrum, users of low-energy glass reported fewer sleep difficulties and higher daylight satisfaction. This reveals the complex relationship that building glass selection needs to balance energy efficiency and health effects. Although this work is based on simulation optimization, through multi-objective GA, it can achieve an adjustable balance between different performance objectives and provide specific parameters applicable to practical design. It provides theoretical guidance and an optimization framework for similar field studies, with strong promotion potential. In conclusion, this work incorporates energy consumption, carbon emissions, and health performance into optimization objectives simultaneously and considers the different needs of windows with different orientations. Compared with existing studies, the method of this work is more systematic and scalable. It provides a scientific basis for high-performance glass design while offering a feasible technical path for low-carbon and healthy building design. This has important theoretical value and practical significance for achieving building energy conservation, carbon emission reduction, and improving the residential health environment.

It is worth noting that the optimization model of this work does not directly simulate the spectral characteristics of glass; however, its optimization results—seeking the optimal combination between lower SHGC and higher VT—are essentially highly consistent with the design concept of high-performance spectrally selective glass. By applying a special functional coating on its surface, spectrally selective glass can selectively transmit the visible light band to ensure natural daylighting and vision. Meanwhile, it can effectively reflect or absorb the near-infrared band in solar radiation to reduce unnecessary solar heat gain. This spectral regulation capability allows it to break the positive correlation usually presented by SHGC and VT parameters in traditional glass. Thus, a practical and feasible technical solution can be provided for realizing the parameter optimization path advocated by this work. For example, the optimization results suggest that south-facing windows adopt a relatively higher SHGC to facilitate passive heat gain in winter, while maintaining a higher VT to ensure daylighting. This parameter combination is exactly a typical feature of spectrally selective Low-E glass. Its coating design allows a large amount of visible light to pass through but has a high reflectivity for near-infrared radiation. Thus, this allows more solar radiation heat to enter the room in winter while maintaining excellent daylighting performance throughout the year. For east and west-facing windows, the optimization results show a lower SHGC requirement to resist the severe summer overheating risk caused by low-angle sunlight. At this time, dynamic glass with stronger solar radiation regulation capability or spectrally selective glass with a lower shading coefficient may be ideal carriers for achieving this optimization goal. The simulation of this work is based on comprehensive SHGC and VT parameters and does not deeply decompose spectral details. However, previous studies have confirmed that different bands in short-wave solar radiation have different impacts on building thermal comfort, and near-infrared radiation is the main factor causing an increase in human thermal sensation. Therefore, using spectrally selective glass, by filtering near-infrared radiation, can generate a lower radiant heat load than ordinary glass under the premise of maintaining the same illuminance level. Thus, it can improve the health performance of the indoor thermal and humid environment at a deeper level.

In conclusion, the multi-objective optimization results of this work demonstrate the great potential and advantageous directions of applying spectrally selective glass in hot-summer and cold-winter regions from the perspective of building performance requirements. Future research could utilize the spectral transmittance curve of glass as a more precise optimization variable. This approach would enable more direct coupling between material optical properties and overall building environmental performance during the design stage. This provides more precise guidance for promoting the design of next-generation high-performance building envelopes.

## 5 Conclusion

Through the GA-based multi-objective optimization study of residential building glass in summer-hot and winter-cold regions, this work reaches the following conclusions: (1) In the hot-summer and cold-winter climate conditions of Hangzhou, the optimization of window glass can significantly improve building energy efficiency and reduce carbon emissions. The optimized glass thermal transmittance, SHGC, and VT all show considerable improvements, particularly for South-facing windows, where the optimization of thermal transmittance and SHGC is most significant. This indicates that South-facing windows have better thermal performance and solar radiation heat utilization efficiency. (2) The optimization scheme effectively reduces the building’s operational energy consumption and carbon emissions. The optimized building’s operational energy consumption decreases from 50.95 kWh/(m^2^·a) to 40.26 kWh/(m^2^·a), a reduction of 20.98%; carbon emissions also drop from 2,623 kgCO₂e/m^2^ to 2083 kgCO₂e/m^2^, a reduction of 20.57%, fully verifying the improvement of building energy efficiency and environmental benefits from the optimization scheme. (3) In terms of improving indoor health performance, the optimized window glass effectively enhances the indoor thermal-humidity and light environment. The proportion of healthy indoor time increases from 34.46 to 40.9%, particularly excelling in illuminance uniformity, illuminance intensity, and glare reduction, improving the comfort of the occupants. This means that the time residents spend in thermally and visually comfortable environments throughout the year is substantially extended. This goes beyond the scope of traditional building energy conservation and provides quantifiable technical paths and case support for proactively promoting public health through architectural design. In summary, the findings demonstrate that optimizing glass performance parameters can effectively improve building energy efficiency and environmental benefits while also enhancing the health environment for residents. It can achieve multi-objective optimization in terms of energy efficiency, carbon emissions, and health performance. Therefore, it is recommended that, in building design for summer-hot and winter-cold regions, South-facing windows be prioritized, and reasonable glass performance optimization be applied according to the characteristics of windows in different orientations to achieve comprehensive optimization in building design. This work provides a scientific basis for architects to design residential buildings in hot-summer and cold-winter regions. It suggests that priority should be given to optimizing the glass performance of south-facing windows during the design process to improve energy efficiency and health performance. At the same time, policymakers can incorporate relevant optimization standards into building energy conservation codes to promote the popularization and application of high-performance glass and advance the development of low-carbon buildings and healthy cities.

Although this work has achieved significant results in optimizing window glass performance for residential buildings in summer-hot and winter-cold regions, there are still certain limitations. First, the work primarily focuses on the climate conditions in Hangzhou and has not explored the adaptability to other regions. Hence, future research could expand to optimization schemes for different climate zones and building types to verify their universality and adaptability. The 18.69% increase in the proportion of healthy time is a value based on simulation results and has not undergone statistical significance testing. The result reflects the predicted trend under the set model parameters and environmental conditions. In subsequent studies, more measured data can be used for verification to enhance the reliability of the conclusions. Moreover, the optimization results are based on short-term simulations and lack long-term field tracking data, so the impact of glass performance optimization on long-term building operations has not been fully reflected. Future research could further improve the optimization model through long-term monitoring data and actual building feedback, and evaluate its long-term benefits. In addition, this work does not fully consider the interactive effects of building structure, interior layout, and other factors on the glass optimization results. Future studies could conduct multi-dimensional optimization research in more complex building environments. Through these further studies, more comprehensive and in-depth technical support could be provided for low-carbon buildings and healthy city planning. Specifically, future research can conduct 12–24 months of monitoring in several representative residential units. Data, such as indoor and outdoor temperature and humidity, illuminance, CO_2_ concentration, sub-item energy consumption of air conditioners and lighting, and glass surface temperature, are collected. Bayesian calibration and data assimilation methods regularly update the simulation model. These methods quantify the long-term energy efficiency, health benefits, and life-cycle carbon emission changes of glass optimization in different seasons and operating conditions. Future research could enhance the applicability and robustness of conclusions by incorporating additional variables into the optimization decision space. These may include building geometry parameters like window-to-wall ratio and orientation, envelope heat capacity, internal heat loads, shading and ventilation strategies, HVAC performance characteristics, and usage behavior patterns. First, key factors are screened through global sensitivity analysis. Then, multi-objective GA combined with surrogate models such as Kriging/polynomial chaos and multi-fidelity simulation is used to conduct multi-dimensional optimization and robustness analysis. Finally, the Pareto fronts under different climate zones are compared to provide cross-climate design recommendations. At the same time, a life-cycle cost model covering initial investment, operation and maintenance, and potential health benefits is constructed. Meanwhile, this model is integrated into the optimization framework with economic objectives to evaluate the economic feasibility of different optimization schemes.

## Data Availability

The original contributions presented in the study are included in the article/supplementary material, further inquiries can be directed to the corresponding author.
